# Spatial variation in hyperthermia emergency department visits among those with employer-based insurance in the United States – a case-crossover analysis

**DOI:** 10.1186/s12940-015-0005-z

**Published:** 2015-03-04

**Authors:** Shubhayu Saha, John W Brock, Ambarish Vaidyanathan, David R Easterling, George Luber

**Affiliations:** Climate and Health Program, Division of Environmental Hazards and Health Effects, National Center for Environmental Health, Centers for Disease Control and Prevention, Atlanta, 30341 GA USA; Departments of Chemistry and Environmental Studies, Warren Wilson College, PO Box 9000, CPO 6066, Asheville, 28815 NC USA; Environmental Health Tracking Branch, Division of Environmental Hazards and Health Effects, National Center for Environmental Health, Centers for Disease Control and Prevention, Atlanta, 30341 GA USA; National Climatic Data Center, Asheville, 28801 NC USA

**Keywords:** Case-crossover, Extreme heat, Hyperthermia, Meta-analysis, Spatial risk

## Abstract

**Background:**

Predictions of intense heat waves across the United States will lead to localized health impacts, most of which are preventable. There is a need to better understand the spatial variation in the morbidity impacts associated with extreme heat across the country to prevent such adverse health outcomes.

**Methods:**

Hyperthermia-related emergency department (ED) visits were obtained from the Truven Health MarketScan® Research dataset for 2000-2010. Three measures of daily ambient heat were constructed using meteorological observations from the National Climatic Data Center (maximum temperature, heat index) and the Spatial Synoptic Classification. Using a time-stratified case crossover approach, odds ratio of hyperthermia-related ED visit were estimated for the three different heat measures. Random effects meta-analysis was used to combine the odds ratios for 94 Metropolitan Statistical Areas (MSA) to examine the spatial variation by eight latitude categories and nine U.S. climate regions.

**Results:**

Examination of lags for all three temperature measures showed that the odds ratio of ED visit was statistically significant and highest on the day of the ED visit. For heat waves lasting two or more days, additional statistically significant association was observed when heat index and synoptic classification was used as the temperature measure. These results were insensitive to the inclusion of air pollution measures. On average, the maximum temperature on the day of an ED visit was 93.4^o^F in ‘South’ and 81.9^o^F in the ‘Northwest’ climatic regions of United States. The meta-analysis showed higher odds ratios of hyperthermia ED visit in the central and the northern parts of the country compared to the south and southwest.

**Conclusion:**

The results showed spatial variation in average temperature on days of ED visit and odds ratio for hyperthermia ED visits associated with extreme heat across United States. This suggests that heat response plans need to be customized for different regions and the potential role of hyperthermia ED visits in syndromic surveillance for extreme heat.

**Electronic supplementary material:**

The online version of this article (doi:10.1186/s12940-015-0005-z) contains supplementary material, which is available to authorized users.

## Introduction

Recent climate assessments indicate more frequent, more intense, and longer-lasting heat waves for most of the United States [1,2]. Though there is evidence suggesting a declining trend in heat-related mortality in the country [3,4], an aging population and inadequate use of residential air conditioning imply that specific sections of the population will continue to remain vulnerable to the projected increase in extreme heat resulting in a large burden of associated adverse health outcomes [5,6]. While the spatial variation in mortality risk from heat waves is shown to vary across different parts of United States [7], little is known about the spatial variation in risk of morbidity outcomes from extreme heat. Since adverse health impacts from extreme heat are preventable [8], public health agencies may need to consider a range of localized health outcomes for syndromic surveillance as part of designing heat response plans [9-12]. Hyperthermia is a direct physiologic impact from exposure to external heat that impair the thermoregulation mechanisms in the body leading to severe consequences [13]. Syndromic systems based on hyperthermia-related emergency department (ED) visits have been found to be effective in early detection of health impacts during heat waves [14]. During the 2006 heat wave in California, 13% (2134 out of 16166) of the estimated excess ED visits were reported for hyperthermia [15]. A report found that 80% of all hyperthermia-related hospital admissions began in an ED setting, supporting the need to further examine impacts of ambient heat on ED visits [16]. Yet, in the search of the literature on heat and morbidity outcomes [17,18], only one study was found that specifically examined measures of ambient heat and the risk of a hyperthermia-related ED visit [19].

This study therefore examines the associations between measures of ambient heat and hyperthermia-related ED visits during April through September from 2000–2010. Novel patient-level health data was obtained from a health insurance database across multiple U.S. metropolitan statistical areas (MSA). The association between hyperthermia-related ED visit with various measures of ambient heat as found in previous epidemiologic studies was examined – maximum temperature, heat index and Spatial Synoptic Classification [7,19-22], since there is no unanimity on any one measure being superior to others [23]. The additional health impact associated with a heat wave [24] was estimated. A case-crossover design as used in recent studies of heat morbidity [19,21,25] was used to estimate the odds ratio of hyperthermia-related ED visit while controlling for the impact of ambient PM_2.5_ and ozone concentrations. Finally, the geographic variation in the odds ratio of ED visit was examined by U.S. national climatic regions [26] and latitude zones [27] using random-effects meta-analysis.

## Methods

### Health data

For the years 2000–2010, health data from the Truven Health MarketScan® Research database including (i) Commercial Claims and Encounters (CCAE) and (ii) Medicare Supplemental and Coordination of Benefits were obtained. The database is a large convenience sample representative of the US population with employer-based health insurance. The large sample size provides a rare opportunity to examine geographic variation in health effects for low-prevalence conditions like hyperthermia. The database captures de-identified patient-level episode with age, gender, date of healthcare service, county of residence, county of emergency department visited, and a list of diagnoses codes based on the ninth revision of the International Classification of Disease (ICD-9-CM). From all available ED visits in the database, patients were included in the study with (i) an ED visit with a diagnosis of hyperthermia (ICD-9-CM = 992.0-992.9 or injury code = E900.0) anywhere in the record; (ii) episodes occurring between April 1 and September 30 in a calendar year; (iii) same county of residence and the county where the ED was located. Patients with injuries due to excessive heat of man-made origin (ICD-9-CM E900.1) were not included. All five-digit county Federal Information Processing Standard (FIPS) information was converted to a MSA for each hyperthermia-related ED visit. Each of the 141 MSAs for which health data was available had a weather-station located within the jurisdiction.

### Measures of ambient heat

Hourly weather data was obtained from the land-based weather stations in these 141 MSAs for each day of the year for 2000–2010 (Integrated Surface Hourly, National Climatic Data Center). Climate Normal information was obtained for each weather station for each day of the year for the period 1980–2010 [28]. Records for weather stations located at major urban airports were used and all stations had less than 5% of days with missing data for the 30 year period. Within each day, missing data were interpolated for up to 4 sequential missing data points by the hour. Days with more than 4 sequential missing data points were excluded from further analyses. The highest recorded temperature among the 24 hourly records (midnight to midnight) was used for daily maximum temperature. The Steadman heat index [29] was used except when temperature or humidity was below 70°F and 40% respectively, the index was set to the dry-bulb air temperature [30]. Daily spatial synoptic classification (SSC) of weather was available for only 107 of these MSAs (http://sheridan.geog.kent.edu/ssc.html). SSC information was available for 90% of the cases. An indicator variable was created to designate a day as extremely hot if the SSC was characterized by the following air mass types - DT,MT+, MT++ for locations in the East of Rockies; DT, MT, MT+, MT++ for locations in the West of Rockies (based on personal communication with Dr. Scott Sheridan). Any day with missing SSC information was not included in the analysis. A heat wave was defined as a block of two or more consecutive days leading to the ED visit when daily maximum temperature or heat index was above the 95^th^ percentile of the 30-year normal for the station [24]. For the SSC, any day that was part of two or more consecutive days with the SSC indicator of extreme heat was deemed to be part of a ‘heat wave’.

### Air quality data

PM_2.5_ daily maximum concentrations (μg/m^3^) and daily maximum 8-hour average ozone concentrations (ppb) were obtained from the EPA’s Data Mart (http://www.epa.gov/ttn/airs/aqsdatamart/) for monitors designated as Federal Reference Methods (FRM). A daily MSA level dataset was created by retaining the maximum concentration among all monitors within the MSA for each monitored day. Although ozone monitors operate on a daily basis during the summer months, PM monitors typically operate on a once-every-third-day schedule [31]. Since ozone and PM_2.5_ were simultaneously factored in the analysis, information on both pollutants was available for about 35% of the cases.

The health, temperature and air pollution datasets were them merged by MSA and date. In the final dataset, days that were designated as U. S. federal holidays during the study period were identified.

### Statistical analysis

A case-crossover design was used for the analysis [32]. Given the ED visit day for each patient, control days were identified using time-stratified approach [33]. In the time-stratified approach, each month was a priori divided into two halves, (i) days 1–15 and (ii) days 16-end of month, and control day(s) were selected on the same weekday as the case within the same half of the month.

### Assessment of odds ratio of ED visit

Conditional logistic regressions (using SAS software ®) was used to assess the odds ratio of hyperthermia-related ED visit and 3 different temperature metrics using the entire dataset. Separate models were estimated for exposures on day_0_ to day_−6_ and the smallest Akaike Information Criteria (AIC) was obtained for day_0_ for all three temperature metrics. In subsequent analysis, day_0_ temperature metric was used. In a series of regressions, the odds ratio for the measure of ambient heat alone was estimated first (Model A). Then, the heat wave indicator was added to assess if there was any additional heat wave effect (Model B). To model B, measures of air pollution and an indicator for U.S. federal holidays were added (Model C) to see if the association between temperature metric and ED visit was sensitive to those factors. Since estimates of PM_2.5_ and ozone were missing for some days, the sample size in Models C and D were smaller than those in Models A and B. Finally, Model B was replicated using the smaller dataset with non-missing air pollution measures (Model D) to check for consistency in results between the pooled sample and the sub-sample with complete air pollution information.

### Estimation of geographic variation in temperature profile and odds ratio for ED visits

Each MSA was classified into one of the (i) nine US climatic regions and (ii) eight latitude categories spanning the continental U.S. (<30°N, >42°N, and six categories in between in 2° intervals). The average temperature profile (using daily maximum temperature, maximum heat index and SSC) were calculated for the days of ED visit and compared with the ‘control’ days by the climate regions. Some areas of the country are sparsely represented in the health database. Since the estimate of the odds ratio for some MSAs with low number of cases of hyperthermia ED visits could be statistically unreliable, estimates were pooled together geographically to derive more statistically robust results. Random-effects meta-analysis was used to combine the MSA-specific odds ratios by the climate regions and latitude zones. An a priori decision was made to include MSAs which had at least 50 cases of ED visits in the dataset. Comprehensive Meta-analysis Software ® was used to perform this task.

### Sensitivity analyses

For selecting control days for the case-crossover analysis, the symmetric bidirectional approach was also used. Following this approach, control days were chosen in a two-week window before and after the case day for the same weekday as the ED visit. The results were comparable to those obtained using the time-stratified approach. An alternative definition of a heat wave - 2 or more consecutive days above the 98^th^ percentile of maximum temperature – yielded comparable results. To check if the results were sensitive to the case definition of hyperthermia, regression results using the sub-sample with only principal diagnosis of hyperthermia produced similar results compared to the entire sample.

## Results

### Characteristics of study population

The dataset comprised of 11,031 episodes of hyperthermia-related ED visits spanning 141 MSAs. The characteristics of the study population are presented in Table 1. A majority (61%) of the patients belonged to the 25–64 years age group, followed by the patients in the 6–18 years group (22%). The age distribution of all individuals included in the MarketScan database for the study years was similar. 68% of the patients were male. 80% of the ED visits occurred in June, July and August. Specifically, 52% of the ED visits within the 13–18 year age group of junior- and high school-aged children were in August and September (Additional file 1: Table S1). 82% of the patients lived either in the South, Southeast, Central or Northeast climate regions. Comparing the percentage of all individuals in the MarketScan database with the hyperthermia ED cases by the climate regions, hyperthermia ED cases were higher from the South and Southeast, and lower from the Northeast and West. A relatively higher percentage of ED visits occurred on Saturdays (19%) compared to the average on other days of the week (~14%).

**Table 1:** **Descriptive statistics of patients with hyperthermia-related emergency department visit 2000–2010 (N = 11031)**

**Age**	**N**	**%**	**Month**	**N**	**%**	**Latitude (** ^**o**^ **N)**	**N**	**%**
<= 5 years	149	1	April	354	3	<30	1107	10
6-12 years	561	5	May	1009	9	<=30 & <32	666	6
13-18 years	1918	17	June	2505	23	<=32 & <34	3042	28
18-24 years	816	7	July	3089	28	<=34 & <36	1513	14
25-50 years	4502	41	August	3145	29	<=36 & <38	864	8
51-64 years	2220	20	September	929	8	<=38 & <40	1571	14
65+ years	865	8				<=40 & <42	1514	14
			US Climate region*			> = 42	754	7
Gender			Central	2249	20			
Female	3502	32	East North Central	531	5	Weekday		
Male	7529	68	Northeast	1239	11	Sunday	1549	14
			Northwest	83	1	Monday	1515	14
Hyperthermia DX			South	3253	29	Tuesday	1589	14
Primary	9262	82	Southeast	2454	22	Wednesday	1498	14
Secondary	1727	16	Southwest	340	3	Thursday	1419	13
			West	803	7	Friday	1402	13
			West North Central	79	1	Saturday	2059	19

*Climate regions are comprised of the following states:

Central: KY, IL, IN, MO, OH, TN, WV;

East North Central: IA, MI, MN, WI;

Northeast: CT, DE, ME, MD, MA, NH, NJ, NY, PA, RI, VT;

Northwest: ID, OR, WA;

South: AR, LA, KS, MS, OK, TX;

Southeast: AL, FL, GA, NC, SC, VA;

Southwest: AZ, CO, NM, UT;

West: CA, NV;

West North Central: MT, NE, ND, SD, WY.

### Assessment of odds ratio of ED visit

For the different measures of ambient temperature, the temperature measures on day_0_ were most strongly correlated with the likelihood of ED visit (smallest Akaike Information Criteria across all the models) (Additional file 1: Table S2). In all subsequent analyses, the same day measure of ambient heat was used. These results concurred with findings in other studies where same-day temperature measures were most influential for heat-related morbidity [19].

The odds ratio of ED visit for maximum temperature and heat index remain unchanged with inclusion of other variables (Table 2). For example, a 1°F increase in maximum temperature was associated with an odds ratio of 1.15 (95% confidence interval: 1,14, 1,16) in model A. Inclusion of the heat wave indicator variable in model B or the air pollution variables in model C did not change the odds ratio estimates. In contrast, when the SSC is used, the association attenuates when other covariates are included in the model. Evidence for an additional heat wave impact (using the 95^th^ percentile threshold) is observed when using heat index (odds ratio = 1.46, 95% confidence interval: 1.26, 1.70) and SSC indicator (odds ratio = 1.25, 95% confidence interval: 1.09, 1.44) as the temperature metric. The associations of the air pollution variables remain consistent across the models with a small positive magnitude. Since the estimates for maximum temperature were similar across the model specifications, model A with the complete sample of observations was used to calculate MSA-specific odds ratios.

**Table 2:** **Regression results for time-stratified case-crossover analyses (pooled data 2000–2010)**

	**Model A**		**Model B**		**Model C**		**Model D**	
	**OR**	**95% CI**	**OR**	**95% CI**	**OR**	**95% CI**	**OR**	**95% CI**
Maximum temperature °F	1.15	1.14, 1.16	1.15	1.14, 1.16	1.15	1.14, 1.17	1.16	1.15, 1.17
Heat wave indicator^a^			1.13	0.96, 1.33	1.14	0.84, 1.55	1.15	0.84, 1.54
PM_2.5_ concentration (g/m^3^)					1.02	1.01, 1.03		
Ozone concentration (ppb)					0.99	0.98, 1.01		
Holiday indicator^b^					1.23	0.82, 1.88		
Model AIC	14833		14830		4972		4983	
Cases	11031		11031		3756		3756	
Control days	13774		13774		4655		4655	
Maximum heat index	1.12	1.11, 1.21	1.11	1.11, 1.12	1.11	1.10, 1.12	1.12	1.11, 1.13
Heat wave indicator^a^			1.46	1.26, 1.70	1.47	1.09, 1.98	1.58	1.17, 2.12
PM_2.5_ concentration (g/m^3^)					1.01	1.01, 1.02		
Ozone concentration (ppb)					1.01	1.00, 1.01		
Holiday indicator					1.25	0.82, 1.90		
Model AIC	14902		14879		4962		5007	
Cases	11031		11031		3756		3756	
Control days	13774		13774		4655		4655	
Spatial synoptic classification	2.56	2.39, 2.74	2.14	1.89, 2.43	1.68	1.36, 2.08	1.99	1.61, 2.45
Heat wave indicator^c^			1.25	1.09, 1.44	1.32	1.04, 1.65	1.32	1.05, 1.67
PM_2.5_ concentration (g/m^3^)					1.03	1.02, 1.04		
Ozone concentration (ppb)					1.01	1.01, 1.02		
Holiday indicator					1.27	0.85, 1.91		
Model AIC	14996		14986		5349		5621	
Cases	9978		9978		3672		3672	
Control days	12442		12442		4537		4537	

Maximum daily temperature, maximum daily heat index and SSC correspond to day of ED visit (day_0_). Model B indicates if there is an additional ‘heat wave’ duration effect. Since PM_2.5_ and Ozone concentrations are not available for all days, the sample sizes for Models C & D are smaller. Comparison of Models B & D indicate if the restricted sample used in Model D (observations with air pollution variables) produce different effect estimate than those obtained using the full sample in Model B.

^a^Heat wave indicator denotes any day that is part of 2 or more consecutive days when maximum temperature was above the 95^th^ percentile for the MSA.

^b^Holiday indicator denotes a U.S. Federal holiday. ^c^Heat wave indicator denotes any day that is part of 2 or more consecutive days when SSC indicator of extreme heat.

### Geographic variation in temperature profile and odds ratio for ED visits

**Figure 1:**
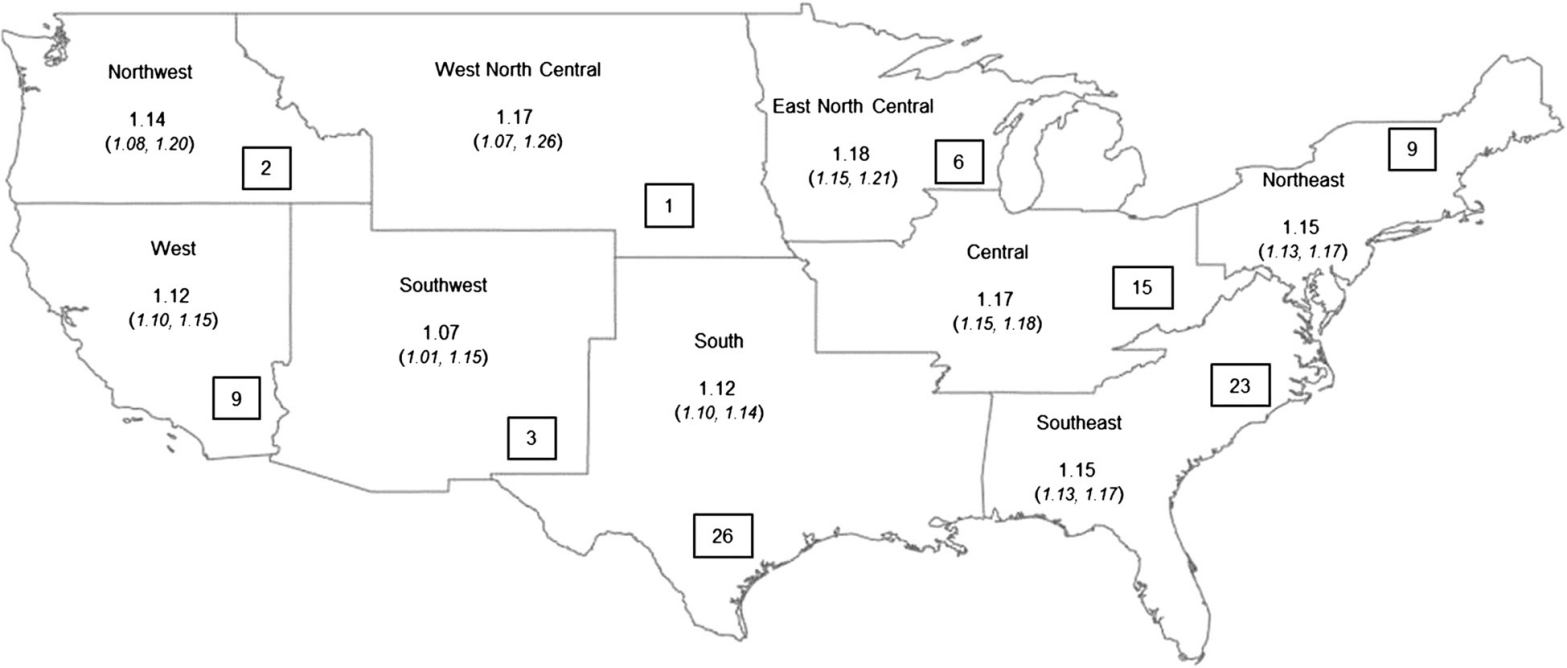
**Results from the random effect meta-analysis of odds ratios of hyperthermia-related ED visit associated with maximum temperature grouped by US climate regions.** Any MSA with less than 50 observations were excluded. The number in the boxes on top show the number of MSAs included in the meta-analysis for each latitude category. Hyperthermia data on ED visit was obtained from MarketScan research database for 2000–2010.

**Figure 2:**
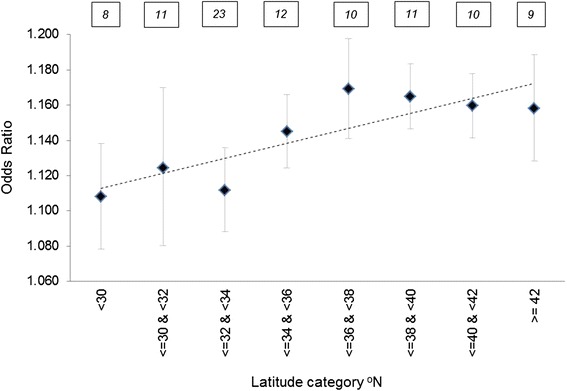
**Results from the random effect meta-analysis of odds ratios of hyperthermia-related ED visit associated with maximum temperature grouped by latitude categories.** Any MSA with less than 50 observations were excluded. The number in the boxes on top show the number of MSAs included in the meta-analysis for each latitude category. Hyperthermia data on ED visit was obtained from MarketScan research database for 2000–2010.

For all three temperature metrics, temperature profiles on the day of the ED visit varied across regions. For example, maximum temperature values on the day of an ED visit were higher in the southern climatic regions compared to the northern regions on average, with a range of 93.4°F in ‘South’ and 81.9°F in the ‘Northwest’ (Table 3). Temperature profiles on days of the ED visit were hotter compared to the ‘control’ days across all climate regions. For example, among the MSAs in the ‘Central’ region, the maximum temperature on the day of an ED visit was 4.9°F higher on average compared to the ‘control’ days. However, this pattern was not uniform across regions, as case days in the north were much hotter compared to the south on average. This spatial variation in temperature profiles on days of ED visit led to the examination of the geographic variation in the odds ratio of hyperthermia-related ED visits. The odds ratio for hyperthermia-related ED visit was estimated for 94 MSAs individually which had at least 50 cases of ED visits each in the dataset (Additional file 1: Table S3). Then random-effects meta-analysis was used to derive estimates for each of the U.S. climatic regions and latitude zones. Test of heterogeneity [34] showed that the odds ratios derived for the different geographic groups of the MSAs were significantly different from each other (I^2^ statistic = 31.1, P < 0.003). The odds ratios for each climatic region were statistically significant, and varied from 1.07 [95% confidence interval: 1.01, 1.15] in the Southwest region to 1.18 [95% confidence interval: 1.15, 1.21] in the East North Central region (Figure 1). The odds ratios for each latitude category were statistically significant, and varied from 1.11 [95% confidence interval: 1.07, 1.14] in the southernmost MSAs to 1.17 [95% confidence interval: 1.14, 1.19] in the northernmost MSAs (Figure 2). Odds ratio of ED visits were relatively higher in the northern MSAs (within 36°N and 42°N latitudes), compared to those below 36°N. Summary results from the meta-analyses using heat index and SSC produced very similar spatial patterns which are presented in the (Additional file 1: Table S4).

**Table 3:** **Average temperature profile on days with hyperthermia ED visit (case day) compared with control days by us climate regions**

	**Case day**			**Control day**		
**Climate region**	**Average maximum temperature**	**Average maximum Heat Index**	**% of days indicated as ‘extremely hot’ by SSC**	**Average maximum temperature**	**Average maximum Heat Index**	**% of days indicated as ‘extremely hot’ by SSC**
Central	88.5	93.3	37	83.6	88.7	17
East North Central	85.4	89.8	39	78.8	84.4	11
Northeast	87.4	90.6	43	80.6	85.4	12
Northwest	81.9	81.2	43	71.4	79.3	13
South	93.4	97.0	38	91.3	95.2	29
Southeast	90.4	95.1	32	87.7	92.4	19
Southwest	90.6	84.6	70	89.0	84.1	66
West	87.2	81.8	46	82.3	80.7	30
West North Central	89.8	93.4	39	83.6	86.1	17

## Discussion

A unique patient-level insurance claims database was combined with different measures of daily ambient heat and analyzed using a case-crossover study design to estimate odds ratio of hyperthermia-related ED visit. Our analyses show a gradient in the impacts of heat across the continental United States - higher odds ratios of hyperthermia ED visits were estimated in the central and the northern parts of the country compared to the south and southwest. This finding aligns with a previous study on heat mortality in 48 cities in the United States [7] that found the risk of mortality to be higher in the Northeast and Midwest compared to the South. Patients in the northern parts of the country visit the ED with a hyperthermia diagnosis on days with lower daily maximum temperatures than in southern parts on average. People living in higher latitudes have previously been found to be more susceptible to extreme high temperatures [11]. Further, our results suggest that patients in the northern parts may be more sensitive to unusually hot days, as the temperature difference between ED visit days and control days was higher on average in northern parts compared to southern parts. The potential of greater tolerance and adaptability among people in the southern regions of US is also mentioned in analysis of heat-related 911 emergency dispatches in Phoenix and Chicago [35]. While different heat metrics produced different magnitudes of odds ratios for hyperthermia ED visits, the spatial pattern in regional estimates of these odds ratios were similar. The range of temperature values associated with mortality across different parts of the US [7] however were different compared to the temperature profile we find on days of hyperthermia ED visits in our data.

Our finding of the strongest association of ambient heat on the day of an ED visit and a significant association up to 3 days prior is similar to that found in previous studies [18]. While several studies have examined the impact of extreme temperature on morbidity, few specifically studied hyperthermia as an outcome. The risk estimates of hyperthermia ED visits from extreme heat found for California [19] (% excess risk per 10°F was 393.3) is similar to what was found for the Western states of California and Nevada in this study (% excess risk for 10°F was 210.5). Additionally, these results suggest that middle and high school aged youth have a higher frequency of hyperthermia-related ED visits in August and September. The literature supports these findings with evidence on increased risks among adolescents playing outdoor sports and thus active youth are deemed to be among the at-risk population during heat waves [36,37]. The association between heat and ED visit was not found to be sensitive to inclusion of air pollution factors in the analysis. While PM_2.5_ was found to have no effect on the association between apparent temperature and emergency department visit [19], the effect of heat wave on mortality was found to be higher on high ozone and PM_10_ days [38]. In a review article on morbidity impacts of heat [18], little agreement was found on potential confounding or modification role of air pollution on heat-related morbidity.

The study has several limitations. The health data used in this study include only individuals with employer-based health insurance or Medicaid living in urban areas, thus limiting the generalizability of the results to the broader US population. As the health risk from excess heat is considerable among the elderly, the uninsured [16] and those living in rural regions [39], the regional estimates derived in this analysis could represent a lower bound. Using the online query system provided by the Healthcare Cost and Utilization Project (http://hcupnet.ahrq.gov/), an average of 50% of patients with hyperthermia-related ED visit in the US between 2006 and 2010 were found to have had health insurance through private sources or Medicaid. MarketScan also slightly over represents the ‘Central’ and ‘Southern’ climatic regions and under represents the ‘Northeast’ as compared to the distribution of the overall population. The fact that 71% of the hyperthermia ED visits used in this study were from the ‘Central’, ‘South’ and ‘Southeast’ climatic regions could be a result of this artifact of the database. However, national rates of hyperthermia-related ED visits were found to be highest in the South and Midwest regions [40] in agreement with the finding in this study. While previous studies have examined the impact of extreme heat on a broader suite of diseases [41], this analysis only examines hyperthermia outcome. However, hyperthermia is a direct physiologic manifestation when exposed to high ambient heat and often a part of suite of health conditions included in a heat surveillance system. In the absence of information on individual level exposure to ambient heat for each patient on each day included in the analysis, one of the criteria to be included in the analysis was that the county of patient’s residence and the ED that was visited needed to be the same. This was the only alternative to ensure that the patient was in the same location on the days included in the analysis, and station-based temperature data for each MSA were most likely what they were exposed to. This study only models the linear effect of same day temperature on the health outcome. While this helped establish the utility of health insurance databases in studying health effects of exposure to heat by producing results comparable with results obtained using commonly available hospital records [19], future applications of this dataset will explore the potential non-linear effect of cumulative temperature exposure over a period of time [42].

## Conclusions

Increase in heat-related illness associated with rising temperatures in the future remains a public health concern. A study estimated a two to six-fold increase in excess respiratory illness during warmer summers in 2080–99 compared to 1991–2004 in the state of New York [22]. Establishing syndromic surveillance systems for extreme heat using hyperthermia-related ED visit information has proved to be an effective response strategy [14]. This study shows hyperthermia ED visits to be sensitive to high temperatures across different parts of country, and availability of real-time ED visit information on hyperthermia could be considered an important indicator for heat surveillance systems. Since we find that hyperthermia ED visits were sensitive to extreme heat and the associated temperature profiles were different across regions of the US, public health agencies would need to assess these risks analyzing locally available data and use those findings in the design of comprehensive heat response plans. The Building Resilience Against Climate Effects (BRACE) framework developed by the Centers for Disease Control for public health agencies to prepare for climate change suggest derivation of exposure-outcome relationships to estimate the disease burden for climate-sensitive environmental exposures [43]. The regional exposure-outcome associations derived for hyperthermia add to the available suite of locally-specific estimates to assess heat-related morbidity.

## Additional file

Additional file 1: Table S1. Distribution of hyperthermia-related cases by age group and month. **Table S2.** Results from separate regression models with different temperature metric and different temporal lag. **Table S3.** MSA-specific odds ratio estimates for hyperthermia-related ED visit and maximum temperature on day of ED visit. **Table S4.** Summary of odds ratios for three temperature metrics and hyperthermia ED visit by US climatic regions.
