# Supplementary material for: Spatial variation in hyperthermia emergency department visits among those with employer-based insurance in the United States – a case-crossover analysis
Source: Environ Health. 2015 Mar 4;14:20. doi: 10.1186/s12940-015-0005-z (PMC4352547; doi:10.1186/s12940-015-0005-z)
Supplement: Additional file 1: Table S1. — Distribution of hyperthermia-related cases by age group and month. Table S2. Results from separate regression models with different temperature metric and different temporal lag. Table S3. MSA-specific odds ratio estimates for hyperthermia-related ED visit and maximum temperature on day of ED visit. Table S4. Summary of odds ratios for three temperature metrics and hyperthermia ED visit by US climatic regions. [file 12940_2015_5_MOESM1_ESM.docx]

**Supplemental material**

Table S1. Distribution of hyperthermia-related cases by age group and month

|  | <= 5 years | 6-12 years | 13-18 years | 18-24 years | 25-50 years | 51-64 years | 65+ years |
| --- | --- | --- | --- | --- | --- | --- | --- |
| Apr | 9 | 27 | 56 | 23 | 142 | 65 | 32 |
|  | *6* | *5* | *3* | *3* | *3* | *3* | *4* |
| May | 13 | 75 | 139 | 95 | 412 | 192 | 83 |
|  | *9* | *13* | *7* | *12* | *9* | *9* | *10* |
| Jun | 44 | 136 | 313 | 205 | 1053 | 550 | 204 |
|  | *30* | *24* | *16* | *25* | *23* | *25* | *24* |
| Jul | 42 | 127 | 401 | 237 | 1347 | 660 | 275 |
|  | *28* | *23* | *21* | *29* | *30* | *30* | *32* |
| Aug | 30 | 122 | 753 | 205 | 1243 | 583 | 209 |
|  | *20* | *22* | *39* | *25* | *28* | *26* | *24* |
| Sep | 11 | 74 | 256 | 51 | 305 | 170 | 62 |
|  | *7* | *13* | *13* | *6* | *7* | *8* | *7* |

For each month, the top row indicates count of episodes and bottom row (in italics) indicate the column percentage

Table S2. Results from separate regression models with different temperature metric and different temporal lag

|  | Maximum temperature | | | | Maximum Alternative Heat Index | | | | Spatial Synoptic Classification | | | |
| --- | --- | --- | --- | --- | --- | --- | --- | --- | --- | --- | --- | --- |
|  | Odds Ratio^#^ | 95% CI | | AIC^^^ | Odds Ratio | 95% CI | | AIC | Odds Ratio | 95% CI | | AIC |
| Day -6 | 0.99 | 0.98 | 0.99 | 17472 | 0.99 | 0.98 | 0.99 | 17477 | 0.77 | 0.72 | 0.83 | 15734 |
| Day -5 | 1.00 | 1.00 | 1.00 | 17519 | 1.00 | 1.00 | 1.01 | 17517 | 0.90 | 0.84 | 0.97 | 15782 |
| Day -4 | 1.01 | 1.01 | 1.02 | 17493 | 1.01 | 1.01 | 1.02 | 17480 | 1.06 | 0.99 | 1.13 | 15803 |
| Day -3 | 1.03 | 1.03 | 1.04 | 17342 | 1.03 | 1.02 | 1.03 | 17322 | 1.44 | 1.34 | 1.54 | 15705 |
| Day -2 | 1.06 | 1.05 | 1.06 | 16973 | 1.05 | 1.05 | 1.05 | 16972 | 1.58 | 1.48 | 1.69 | 15614 |
| Day -1 | 1.10 | 1.10 | 1.11 | 16009 | 1.08 | 1.08 | 1.09 | 16080 | 2.19 | 2.04 | 2.35 | 15279 |
| Day 0 | 1.15 | 1.14 | 1.16 | 14833 | 1.12 | 1.11 | 1.12 | 14903 | 2.56 | 2.39 | 2.74 | 14996 |
| Mean (Day 0 to Day -1) | 1.16 | 1.15 | 1.17 | 15000 | 1.13 | 1.12 | 1.13 | 15067 |  |  |  |  |
| Mean (Day 0 to Day -2) | 1.15 | 1.14 | 1.16 | 15422 | 1.13 | 1.12 | 1.13 | 15444 |  |  |  |  |

^^^ Lower AIC value indicate better model fit

Table S3. MSA-specific odds ratio estimates for hyperthermia-related ED visit and maximum temperature on day of ED visit

| MSA | State | | Latitude | | Longitude | NCDC region | | Odds Ratio | 95% CI for Odds Ratio | | | | Controls | | | Cases |
| --- | --- | --- | --- | --- | --- | --- | --- | --- | --- | --- | --- | --- | --- | --- | --- | --- |
| Huntsville | AL | | 34.65 | | 86.58 | Southeast | | 1.31 | 1.11 | 1.54 | | 54 | | | 45 | |
| Mobile | AL | | 30.68 | | 88.25 | Southeast | | 1.27 | 1.01 | 1.59 | | 30 | | | 26 | |
| Montgomery | AL | | 32.3 | | 86.36 | Southeast | | 1.13 | 1.00 | 1.28 | | 42 | | | 33 | |
| Birmingham | AL | | 33.57 | | 86.75 | Southeast | | 1.22 | 1.07 | 1.39 | | 82 | | | 67 | |
| Fayetteville | AR | | 36.06 | | 94.16 | South | | 1.18 | 1.00 | 1.40 | | 34 | | | 29 | |
| Little Rock | AR | | 34.74 | | 92.23 | South | | 1.16 | 1.03 | 1.31 | | 48 | | | 40 | |
| Tucson | AZ | | 32.12 | | 110.93 | Southwest | | 1.17 | 1.02 | 1.33 | | 43 | | | 37 | |
| Phoenix | AZ | | 33.43 | | 112.01 | Southwest | | 1.03 | 0.98 | 1.07 | | 302 | | | 236 | |
| Bakersfield | CA | | 35.43 | | 119.05 | West | | 1.10 | 1.00 | 1.22 | | 29 | | | 23 | |
| Fresno | CA | | 36.77 | | 119.71 | West | | 1.25 | 1.06 | 1.46 | | 39 | | | 28 | |
| Imperial | CA | | 32.83 | | 115.57 | West | | 1.04 | 0.95 | 1.15 | | 39 | | | 29 | |
| Los Angeles | CA | | 34.05 | | 118.25 | West | | 1.16 | 1.11 | 1.22 | | 288 | | | 232 | |
| Oakland | CA | | 37.73 | | 122.31 | West | | 1.16 | 1.08 | 1.24 | | 59 | | | 51 | |
| Riverside | CA | | 33.95 | | 117.25 | West | | 1.09 | 1.05 | 1.13 | | 142 | | | 116 | |
| Sacramento | CA | | 38.7 | | 121.5 | West | | 1.14 | 1.10 | 1.19 | | 201 | | | 153 | |
| San Diego | CA | | 32.73 | | 117.16 | West | | 1.10 | 1.01 | 1.21 | | 65 | | | 48 | |
| Denver | CO | | 39.75 | | 104.86 | Southwest | | 1.10 | 1.01 | 1.19 | | 40 | | | 33 | |
| Hartford | CT | | 41.73 | | 72.65 | Northeast | | 1.20 | 1.06 | 1.36 | | 32 | | | 28 | |
| Washington | DC | | 38.85 | | 77.03 | Northeast | | 1.18 | 1.13 | 1.23 | | 288 | | | 238 | |
| Jacksonville | FL | | 30.5 | | 81.7 | Southeast | | 1.15 | 1.05 | 1.26 | | 75 | | | 67 | |
| Miami | FL | | 25.82 | | 80.26 | Southeast | | 1.11 | 1.00 | 1.25 | | 128 | | | 100 | |
| Orlando | FL | | 28.43 | | 81.38 | Southeast | | 1.18 | 1.03 | 1.36 | | 74 | | | 64 | |
| Tampa | FL | | 27.97 | | 82.53 | Southeast | | 1.17 | 1.02 | 1.35 | | 75 | | | 63 | |
| Albany | GA | | 31.58 | | 84.16 | Southeast | | 1.11 | 0.97 | 1.26 | | 41 | | | 33 | |
| Augusta-Aiken | GA/SC | | 33.37 | | 81.96 | Southeast | | 1.45 | 1.11 | 1.89 | | 31 | | | 24 | |
| Atlanta | GA | | 33.65 | | 84.43 | Southeast | | 1.15 | 1.11 | 1.19 | | 672 | | | 548 | |
| Macon | GA | | 32.7 | | 83.65 | Southeast | | 1.03 | 0.93 | 1.14 | | 61 | | | 48 | |
| Des Moines | IA | | 41.53 | | 93.65 | E. North Central | | 1.13 | 1.04 | 1.24 | | 51 | | | 45 | |
| Decatur | IL | | 39.83 | | 88.86 | Central | | 1.19 | 1.05 | 1.34 | | 36 | | | 29 | |
| Chicago | IL | | 41.9 | | 87.75 | Central | | 1.16 | 1.14 | 1.19 | | 730 | | | 567 | |
| Evansville | IN | | 37.97 | | 87.56 | Central | | 1.17 | 1.07 | 1.29 | | 71 | | | 58 | |
| Indianapolis | IN | | 39.73 | | 86.28 | Central | | 1.23 | 1.15 | 1.32 | | 189 | | | 150 | |
| Wichita | KS | | 33.98 | | 98.48 | South | | 1.10 | 1.02 | 1.19 | | 72 | | | 55 | |
| Louisville | KY | | 38.23 | | 85.73 | Central | | 1.15 | 1.09 | 1.21 | | 157 | | | 129 | |
| Baton Rouge | LA | | 30.53 | | 91.15 | South | | 1.07 | 0.94 | 1.22 | | 35 | | | 32 | |
| Shreveport | LA | | 32.52 | | 93.81 | South | | 1.10 | 0.97 | 1.24 | | 43 | | | 35 | |
| New Orleans | LA | | 29.98 | | 90.25 | South | | 1.14 | 1.02 | 1.27 | | 89 | | | 73 | |
| Boston | MA | | 42.37 | | 71.03 | Northeast | | 1.11 | 1.07 | 1.16 | | 142 | | | 115 | |
| Baltimore | MD | | 39.18 | | 76.66 | Northeast | | 1.17 | 1.11 | 1.22 | | 214 | | | 164 | |
| Grand Rapids | MI | | 42.88 | | 85.51 | E. North Central | | 1.21 | 1.06 | 1.38 | | 32 | | | 24 | |
| Detroit | MI | | 42.23 | | 83.01 | E. North Central | | 1.17 | 1.13 | 1.21 | | 390 | | | 297 | |
| Flint | MI | | 42.97 | | 83.73 | E. North Central | | 1.18 | 1.08 | 1.29 | | 60 | | | 45 | |
| Minneapolis | MN | | 44.83 | | 93.21 | E. North Central | | 1.29 | 1.15 | 1.45 | | 72 | | | 57 | |
| Kansas City | MO | | 39.32 | | 94.58 | Central | | 1.16 | 1.11 | 1.20 | | 290 | | | 235 | |
| St. Louis | MO | | 39.12 | | 90.38 | Central | | 1.15 | 1.10 | 1.21 | | 194 | | | 161 | |
| Gulfport/Biloxi | MS | | 30.4 | | 89.9 | South | | 1.20 | 1.10 | 1.30 | | 128 | | | 98 | |
|  |  | |  | |  |  | |  |  |  | |  | | |  | |
| MSA | | State | | Latitude | Longitude | NCDC region | Odds Ratio | | 95% CI for Odds Ratio | | Controls | | | Cases | | |
| Jackson | MS | | 32.32 | | 90.08 | South | | 1.21 | 1.09 | 1.34 | | 97 | | | 76 | |
| Gulfport/Biloxi | | MS | | 30.4 | 89.9 | South | 1.20 | | 1.10 | 1.30 | 128 | | | 98 | | |
| Jackson | | MS | | 32.32 | 90.08 | South | 1.21 | | 1.09 | 1.34 | 97 | | | 76 | | |
| Charlotte | | NC | | 35.22 | 80.93 | Southeast | 1.15 | | 1.09 | 1.22 | 182 | | | 145 | | |
| Omaha | | NE | | 41.3 | 95.9 | W. North Central | 1.17 | | 1.07 | 1.28 | 55 | | | 40 | | |
| Newark | | NJ | | 40.7 | 74.16 | Northeast | 1.14 | | 1.10 | 1.19 | 198 | | | 165 | | |
| Las Vegas | | NV | | 36.08 | 115.16 | West | 1.13 | | 1.05 | 1.23 | 80 | | | 65 | | |
| Buffalo | | NY | | 42.93 | 78.73 | Northeast | 1.15 | | 1.03 | 1.29 | 38 | | | 29 | | |
| New York | | NY | | 40.77 | 73.96 | Northeast | 1.14 | | 1.11 | 1.18 | 299 | | | 237 | | |
| Cincinnati | | OH | | 39.05 | 84.51 | Central | 1.20 | | 1.11 | 1.30 | 126 | | | 93 | | |
| Cleveland | | OH | | 41.52 | 81.85 | Central | 1.17 | | 1.08 | 1.26 | 78 | | | 59 | | |
| Columbus | | OH | | 40 | 82.88 | Central | 1.18 | | 1.09 | 1.28 | 91 | | | 73 | | |
| Toledo | | OH | | 41.6 | 83.8 | Central | 1.21 | | 1.14 | 1.28 | 177 | | | 131 | | |
| Oklahoma City | | OK | | 35.4 | 97.6 | South | 1.14 | | 1.10 | 1.18 | 377 | | | 306 | | |
| Tulsa | | OK | | 36.2 | 95.9 | South | 1.15 | | 1.09 | 1.22 | 199 | | | 154 | | |
| Portland | | OR | | 45.6 | 122.6 | Northwest | 1.12 | | 1.04 | 1.20 | 37 | | | 29 | | |
| Pittsburgh | | PA | | 40.35 | 80.21 | Northeast | 1.16 | | 1.03 | 1.30 | 38 | | | 28 | | |
| Philadelphia | | PA | | 39.88 | 75.25 | Northeast | 1.20 | | 1.14 | 1.26 | 222 | | | 166 | | |
| Myrtle Beach | | SC | | 33.68 | 78.89 | Southeast | 1.12 | | 1.00 | 1.26 | 52 | | | 44 | | |
| Orangeburg | | SC | | 33.49 | 80.86 | Southeast | 1.17 | | 1.00 | 1.38 | 29 | | | 24 | | |
| Anderson | | SC | | 34.5 | 82.71 | Southeast | 1.16 | | 1.05 | 1.28 | 66 | | | 56 | | |
| Beaufort | | SC | | 32.48 | 80.67 | Southeast | 1.16 | | 1.04 | 1.29 | 59 | | | 48 | | |
| Charleston | | SC | | 32.9 | 79.96 | Southeast | 1.15 | | 1.08 | 1.22 | 197 | | | 162 | | |
| Columbia | | SC | | 33.95 | 81.11 | Southeast | 1.14 | | 1.09 | 1.19 | 290 | | | 232 | | |
| Florence | | SC | | 34.18 | 79.71 | Southeast | 1.10 | | 1.04 | 1.18 | 151 | | | 115 | | |
| Greenville/Spartanburg | | SC | | 34.85 | 82.21 | Southeast | 1.17 | | 1.11 | 1.25 | 186 | | | 158 | | |
| Chattanooga | | TN | | 35.05 | 85.31 | Central | 1.15 | | 1.00 | 1.31 | 35 | | | 26 | | |
| Knoxville | | TN | | 35.82 | 83.98 | Central | 1.18 | | 1.04 | 1.35 | 63 | | | 52 | | |
| Memphis | | TN | | 35.05 | 90 | Central | 1.12 | | 1.07 | 1.17 | 267 | | | 213 | | |
| Nashville | | TN | | 36.12 | 86.68 | Central | 1.27 | | 1.18 | 1.36 | 206 | | | 164 | | |
| Abilene | | TX | | 32.42 | 99.68 | South | 1.08 | | 0.95 | 1.23 | 29 | | | 24 | | |
| College Station | | TX | | 30.58 | 96.55 | South | 1.20 | | 1.02 | 1.42 | 40 | | | 30 | | |
| Corpus Christi | | TX | | 27.77 | 97.5 | South | 1.02 | | 0.90 | 1.16 | 43 | | | 33 | | |
| Longview | | TX | | 32.38 | 94.73 | South | 1.04 | | 0.93 | 1.17 | 31 | | | 25 | | |
| Lufkin | | TX | | 31.23 | 94.8 | South | 0.98 | | 0.84 | 1.14 | 41 | | | 30 | | |
| Temple | | TX | | 31.15 | 97.35 | South | 1.20 | | 1.00 | 1.43 | 44 | | | 36 | | |
| Texarkana | | TX | | 33.43 | 94.05 | South | 1.03 | | 0.95 | 1.12 | 34 | | | 28 | | |
| Tyler | | TX | | 32.37 | 95.26 | South | 1.31 | | 1.10 | 1.57 | 48 | | | 40 | | |
| Victoria | | TX | | 28.81 | 96.91 | South | 1.35 | | 1.07 | 1.69 | 36 | | | 29 | | |
| Waco | | TX | | 31.62 | 97.21 | South | 1.19 | | 1.03 | 1.37 | 48 | | | 41 | | |
| Wichita Falls | | TX | | 37.65 | 97.41 | South | 1.13 | | 1.01 | 1.26 | 31 | | | 24 | | |
| Austin | | TX | | 30.3 | 97.7 | South | 1.07 | | 1.01 | 1.12 | 223 | | | 187 | | |
| Dallas | | TX | | 32.97 | 96.85 | South | 1.11 | | 1.09 | 1.14 | 1208 | | | 991 | | |
| Houston | | TX | | 29.97 | 95.35 | South | 1.10 | | 1.06 | 1.13 | 729 | | | 587 | | |
| Port Arthur | | TX | | 30.58 | 94.01 | South | 1.16 | | 1.01 | 1.33 | 61 | | | 52 | | |
| San Antonio | | TX | | 29.53 | 98.46 | South | 1.13 | | 1.04 | 1.22 | 138 | | | 111 | | |
| Norfolk | | VA | | 36.93 | 76.2 | Southeast | 1.15 | | 1.08 | 1.22 | 126 | | | 96 | | |
| Richmond | | VA | | 37.5 | 77.33 | Southeast | 1.17 | | 1.11 | 1.25 | 131 | | | 105 | | |
| Seattle | | WA | | 47.45 | 122.3 | Northwest | 1.17 | | 1.07 | 1.28 | 48 | | | 40 | | |
| Milwaukee | | WI | | 42.95 | 87.9 | E. North Central | 1.21 | | 1.08 | 1.36 | 43 | | | 33 | | |

Table S4. Summary of odds ratios for three temperature metrics and hyperthermia ED visit by US climatic regions

| US Climate Regions | Maximum temperature | | | Maximum heat index | | | Spatial Synoptic Classification | | |
| --- | --- | --- | --- | --- | --- | --- | --- | --- | --- |
|  | N* | Odds Ratio | 95% CI | N* | Odds Ratio | 95% CI | N* | Odds Ratio | 95% CI |
| Central | 15 | 1.17 | 1.15, 1.18 | 15 | 1.12 | 1.11, 1.13 | 11 | 3.41 | 2.88, 4.02 |
| East North Central | 6 | 1.18 | 1.15, 1.21 | 6 | 1.14 | 1.11, 1.16 | 6 | 6.50 | 4.45, 9.49 |
| Northeast | 9 | 1.15 | 1.13, 1.17 | 9 | 1.13 | 1.11, 1.14 | 8 | 5.58 | 4.45, 6.97 |
| Northwest | 2 | 1.14 | 1.08, 1.20 | 2 | 1.14 | 1.07, 1.20 | 2 | 4.76 | 1.91, 11.8 |
| South | 26 | 1.12 | 1.10, 1.14 | 26 | 1.09 | 1.07, 1.09 | 17 | 1.57 | 1.39, 1.77 |
| Southeast | 23 | 1.15 | 1.13, 1.16 | 23 | 1.11 | 1.09, 1.12 | 18 | 2.02 | 1.73, 2.35 |
| Southwest | 3 | 1.07 | 1.01, 1.15 | 3 | 1.05 | 1.01, 1.09 | 3 | 1.17 | 0.79, 1.73 |
| West | 9 | 1.13 | 1.10, 1.15 | 9 | 1.13 | 1.10, 1.15 | 7 | 2.45 | 1.86, 3.21 |
| West North Central | 1 | 1.17 | 1.07, 1.26 | 1 | 1.13 | 1.05, 1.20 | 1 | 5.12 | 1.66, 15.7 |

N* Number of MSAs that were included in each category to derive the meta-analyzed estimates. Regional estimates derived using few MSAs are statistically unreliable.
